# Competing nucleation of single- and double-layer Guinier–Preston zones in Al–Cu alloys

**DOI:** 10.1038/s41598-021-83920-8

**Published:** 2021-02-24

**Authors:** Hiroshi Miyoshi, Hajime Kimizuka, Akio Ishii, Shigenobu Ogata

**Affiliations:** 1 Department of Mechanical Science and Bioengineering, Graduate School of Engineering Science, Osaka University, Osaka, 560-8531 Japan; 2 Department of Materials Design Innovation Engineering, Graduate School of Engineering, Nagoya University, Aichi, 464-8603 Japan; 3 Center for Elements Strategy Initiative for Structural Materials (ESISM), Kyoto University, Kyoto, 606-8501 Japan

**Keywords:** Metals and alloys, Phase transitions and critical phenomena, Atomistic models, Structural properties

## Abstract

Solid-state precipitation is a key heat-treatment strategy for strengthening engineering alloys. Therefore, predicting the precipitation process of localized solute-rich clusters, such as Guinier–Preston (GP) zones, is necessary. We quantitatively evaluated the critical nucleus size and nucleation barrier of GP zones in Al–Cu alloys, illustrating the precipitation preferences of single-layer (GP1) and double-layer (GP2) GP zones. Based on classical nucleation theory using an effective multi-body potential for dilute Al–Cu systems, our model predicted GP1 and GP2 precipitation sequences at various temperatures and Cu concentrations in a manner consistent with experimental observations. The crossover between formation enthalpy curves of GP1 and GP2 with increasing cluster size determines the critical conditions under which GP2 zones can nucleate without prior formation of GP1 zones. This relationship reflects competing interactions within and between clusters. The results illustrate the underlying mechanisms of competing nucleation between zones, and provide guidance for tailoring aging conditions to achieve desired mechanical properties for specific applications.

## Introduction

Precipitation (or age) hardening is one of the most common approaches used to increase the yield strength and hardness of Al alloys. During the aging of alloys, metastable precipitates with different structures appear and disappear sequentially over time. As a representative example, the precipitation sequence of dilute Al–Cu alloys is generally accepted as^1–3^:

1 $$\begin{aligned} {\text {SSS}} \rightarrow \underbrace{\text {GP1} \rightarrow \text {GP2 (or } \theta ^{\prime \prime } \text {)}}_{\text {GP zones}} \rightarrow \theta ^{\prime } \rightarrow \theta , \end{aligned}$$

where SSS is the supersaturated solid solution after solution treatment and quenching. Nanosized coherent pre-precipitates (or solute clusters), called Guinier–Preston (GP) zones^4,5^, are formed during natural aging and in the early stages of artificial aging. Here, GP1 and GP2 refer to the solute-rich regions formed in the first and second stages of aging, respectively. The GP1 zone is characterized by a single-layer atomic Cu platelet formed in a {100} plane. In contrast, the GP2 zone has a double-layer structure consisting of two GP1 zones, which are typically separated by three Al atomic layers in the face-centered cubic (fcc) Al matrix^6–8^. The GP2 zone is generally responsible for the main hardening process, whereas the metastable $\theta ^{\prime }$ and stable $\theta$ phases are characterized by coarse particles that are typically observed in overaged samples^1,9–11^. Interestingly, it has been suggested that higher peak hardness is provided by the formation of GP2 zones in a matrix already containing GP1 zones, while direct precipitation of either GP2 zones or $\theta ^{\prime }$ phases results in lower peak hardness^1,11^. Therefore, identifying the precipitation process associated with competing nucleation of the two types of GP zones is essential for elucidating the factors that control the structural evolution of solute-rich zones responsible for strengthening behavior.

**Table 1:** Experimental observations by Silcock et al.^11^ of structures first detected during the aging process of Al–Cu alloys at various temperatures and Cu concentrations.

Aging temperature	2 wt% (0.9 at %)	3 wt% (1.3 at %)	4 wt% (1.7 at %)	4.5 wt% (2.0 at %)
383 K (110 $^{\circ }$C)	GP1	GP1	GP1	GP1
403 K (130 $^{\circ }$C)	$\theta ^{\prime }$ or $\theta ^{\prime }$ and GP2 or GP1	GP1	GP1	GP1
438 K (165 $^{\circ }$C)		$\theta ^{\prime }$ and little GP2	GP1 and GP2	
463 K (190 $^{\circ }$C)	$\theta ^{\prime }$	$\theta ^{\prime }$ and very little GP2	GP2 and little $\theta ^{\prime }$	GP2
493 K (220 $^{\circ }$C)	$\theta ^{\prime }$		$\theta ^{\prime }$	$\theta ^{\prime }$
513 K (240 $^{\circ }$C)			$\theta ^{\prime }$	

Silcock et al.^11^ performed the first comprehensive examination of the structural changes in an Al–Cu system. The study covered a wide range of degrees of supersaturation and included X-ray characterization of precipitates directly related to the hardening process for aging at various temperatures. Hardness changes as a function of aging time were observed in the stages associated with the GP1 and GP2 zones. Table 1 lists the experimental results for the structures first detected for various Cu concentrations at aging temperatures from 383 to 513 K. The precipitation sequence under these aging conditions has been consistently observed in many studies^7,12–16^. At lower temperatures, the two stages in the aging curves are separated by a plateau and can be clearly observed. However, this plateau becomes less evident as the aging temperature increases and/or the Cu content is reduced. This is because the formation of GP1 zones is suppressed under these conditions. The full sequence of intermediate stages in the precipitation process, as shown in Eq. (1), tends to be enhanced by a high degree of supersaturation and a low aging temperature. Although state-of-the-art experimental techniques have improved our fundamental understanding of the variations in structure and composition of solute precipitates in Al–Cu alloys^8,16–21^, a detailed explanation of the underlying mechanisms of such competing nucleation between the GP1 and GP2 zones is still lacking.

During the growth of GP zones, the diameter of the zone with the GP1 structure remains quite constant across the plateau of the two-stage hardness curves, and then increases rapidly at the end of the plateau as the GP1 structure changes to the GP2 structure. The average zone diameter was reported as 4–5 nm in an Al-1.7 at %Cu alloy at room temperature^11,22^ and 6–7 nm in an Al-1.94 at %Cu alloy at 373 K^15^. The tendency for the saturation of the zone size has been recognized by many researchers^7,11,13–15,23,24^, although the measured zone diameters are somewhat scattered within a range below 8 nm. To the best of our knowledge, no conclusive explanation exists for the saturation of the GP1 zone size and its abrupt growth after transformation to the GP2 structure.

In this study, we investigated the underlying mechanism of competing nucleation of the GP1 and GP2 zones (i.e., single- and double-layer GP zones, respectively) in Al–Cu alloys. Our aim was to elucidate the effects of changes in the temperature and solute concentration on the preferential nucleation of the two types of GP zones, by considering the energetics of the processes at the atomic level. By exploring the free energy of formation for GP1 and GP2 zones as a function of temperature and solute concentration, we address important questions related to the critical conditions for the zone nucleation observed during the aging process, such as: (i) What mechanism could change the precipitation sequence of the two GP zones? (ii) Under what conditions can GP2 zones nucleate directly without the induction of GP1 zones? (iii) Why is the growth process of GP zones markedly discontinuous? (iv) What determines the upper limit of the size of the GP1 zones? To address these questions, we outline the computational approach and describe our findings from a combination of atomistic and classical nucleation theory (CNT) perspectives.

## Results and discussion

### CNT model of GP-zone formation

We used the CNT approach to obtain the nucleation barrier of GP-zone formation. The nucleation barrier corresponds to the free energy required to drive solute atoms from a solution to form a cluster with a critical nucleus size, at which the forward and reverse driving forces for the growth are balanced. The critical nucleus size is described based on thermodynamic and kinetic aspects. From a thermodynamics standpoint, the nucleation barrier is equivalent to the maximum free energy of formation of the cluster. The free energy of formation of the cluster at a constant temperature and solute concentration is given by:

2 $$\begin{aligned} \Delta G(n) = \Delta H(n) - T\Delta S(n), \end{aligned}$$

where $\Delta H(n)$ and $\Delta S(n)$ are the enthalpy and entropy of formation, respectively, of an *n*-atom solute cluster; and *T* is the absolute temperature. Here, the cluster size is defined as the number of constituent solute atoms *n* (*n* $\ge 1$). Note that $\Delta G(n)$ is defined such that $\Delta G(1) = 0$ (i.e., $\Delta H(1) = \Delta S(1) = 0$), where the initial state is described as the set of isolated ($n = 1$) solute atoms in the matrix. According to the Bragg–Williams approximation, the configurational part of $\Delta S(n)$ for a disk-shaped cluster, such as GP1 and GP2 zones, is practically expressed as^25,26^:

3 $$\begin{aligned} \Delta S(n) = k_{\text {B}}\left[ (n-1)\ln x + \ln W_{\text {rot}}\right] , \end{aligned}$$

where $k_{\text {B}}$ is the Boltzmann constant, *x* is the solute concentration, and $W_{\text {rot}}$ denotes the number of independent configurations due to rotation of the disk-shaped cluster forming on crystallographically equivalent planes. In this study, Eq. (3) was equally applied to GP1 and GP2 clusters, since both have similar planar structures with the same crystallographic characteristics^1,6^.

If *n* is sufficiently large that $\Delta H(n)$ and $\Delta S(n)$ are assumed to be continuous and differentiable with respect to *n*, the maximum $\Delta G(n)$ satisfies $\partial \Delta G(n)/\partial n=0$. Then, the following is obtained:

4 $$\begin{aligned} \left. \frac{\partial \Delta H(n)}{\partial n}\right| _{n=n^{*}_{\text {t}}} = k_{\text {B}}T\ln x. \end{aligned}$$

Equation (4) refers to the condition where the gain in enthalpic contribution to the free energy with an increase in *n* is equal to the decrease in the entropic contribution associated with cluster growth (i.e., removal of a solute atom from an infinitely large matrix). If the temperature and solute concentration are given, *n* satisfying Eq. (4) corresponds to the thermodynamic critical nucleus size $n^{*}_{\text {t}}$, and $\Delta G(n^{*}_{\text {t}})$ is the thermodynamic nucleation barrier.

From a kinetics standpoint, the critical nucleus size is defined as the size at which the attachment rate ($K^+(n)$) of a solute atom to the cluster is balanced by the corresponding detachment rate ($K^-(n)$). If equilibrium conditions are assumed in the nucleation process of *n*-atom solute clusters at the atomic level, the rate of cluster growth should be balanced by the rate of the reverse process, as follows^27^:

5 $$\begin{aligned} K^{+}(n-1)C(n-1) = K^{-}(n)C(n), \end{aligned}$$

where *C*(*n*) is the number density of *n*-atom clusters given by

6 $$\begin{aligned} C(n) = C_0(1)\exp \left( -\frac{\Delta G(n)}{k_{\text {B}}T}\right) , \end{aligned}$$

where $C_0(1)$ is the number density of single solute atoms. Furthermore, $K^+(n)$ is defined as:

7 $$\begin{aligned} K^+(n) = A(n)\exp \left( -\frac{G^+(n)}{k_{\text {B}}T}\right) , \end{aligned}$$

where $G^+(n)$ and *A*(*n*) are the activation free energy and attempt frequency, respectively, for the attachment of a solute atom to the *n*-atom cluster; $G^+(n)$ is approximated by^28,29^:

8 $$\begin{aligned} G^+(n) = G_{\text {a}}+\frac{\Delta G(n+1)-\Delta G(n)}{2}, \end{aligned}$$

where $G_{\text {a}}$ is the free energy of activation for vacancy-assisted migration of a solute atom in the matrix. Then, by assuming $A(n-1) \approx A(n)$ for sufficiently large *n*, the logarithm of the ratio between detachment and attachment rates is described as:

9 $$\begin{aligned} \ln \left( \frac{K^-(n)}{K^+(n)}\right)= & {} \ln \left( \frac{C(n-1)}{C(n)}\frac{K^+(n-1)}{K^+(n)}\right) \nonumber \\= & {} \frac{\Delta H(n+1) - \Delta H(n-1)}{2k_{\text {B}}T}-\ln x. \end{aligned}$$

Here, the sign of the right-hand side of Eq. (9) is determined by the balance between enthalpic and entropic terms. The *n* satisfying $K^-(n) = K^+(n)$ gives the critical nucleus size $n^{*}_{\text {k}}$ giving:

10 $$\begin{aligned} k_{\text {B}}T\ln x= & {} \frac{\Delta H(n^{*}_{\text {k}}+1) - \Delta H(n^{*}_{\text {k}}-1)}{2} \nonumber \\\approx & {} \left. \frac{\partial \Delta H(n)}{\partial n}\right| _{n=n^{*}_{\text {k}}}, \end{aligned}$$

where the final approximation is obtained by the central difference for $\Delta H(n)$ at *n* = $n^{*}_{\text {k}}$. In this case, Eqs. (4) and (10) eventually become equivalent, and the two definitions of the critical nucleus size ($n^{*}_{\text {t}}$ and $n^{*}_{\text {k}}$) correspond to each other. Thus, these two sizes are collectively described as $n^{*}$ in the remainder of this paper.

Finally, by supposing that $\ln W_{\text {rot}}$ is negligible compared with the first term in Eq. (3), which is valid when $n \gg 1$ and/or $x \ll 1$, the nucleation barrier for GP-zone formation is simply described by:

11 $$\begin{aligned} \Delta G(n^{*}) = \Delta H(n^{*})-(n^{*}-1)\left. \frac{\partial \Delta H(n)}{\partial n}\right| _{n=n^{*}}, \end{aligned}$$

where $n^{*}$ is a function of *T* and *x* satisfying Eq. (4) or (10). This indicates that if $\Delta H(n)$ over a wide range of *n* is adequately evaluated by some means, $n^{*}$ and the corresponding $\Delta G(n^{*})$ are obtained by solving these nonlinear equations at various temperatures and solute concentrations.

### Enthalpy of formation of GP1 and GP2 zones

Modern electronic-structure calculations based on density functional theory (DFT) can provide good descriptions of the geometries and energetics of solute clusters and precipitates in Al–Cu systems^30–38^. On the other hand, direct DFT calculations for supercells containing a nucleus of GP1 and GP2 clusters, whose sizes typically reach and even exceed several nanometers, are highly challenging and even infeasible owing to the huge computational costs. In this study, to obtain $\Delta H(n)$ of the GP1 and GP2 zones in Al–Cu alloys, we used an on-lattice effective multi-body potential that considers the interaction between the clusters, which is extension of our previous work^29^.

Using the proposed potential (see Methods section), the enthalpies of formation of GP1 and GP2 clusters were calculated for *n* = 1–112. The discrete set of data with respect to *n* were then fit to a function of the form:

12 $$\begin{aligned} \Delta H(n) = {\left\{ \begin{array}{ll} An-An^{p} &{} {\text {if}} \;\; n \le {\bar{n}} \\ Bn+Cn^\frac{1}{2}+D &{} {\text {otherwise}} \end{array}\right. } , \end{aligned}$$

where *A*, *B*, and *p* (0 < *p* < 1) are fitting parameters; and ${\bar{n}}$ was chosen as 10 and 40 for GP1 and GP2 clusters, respectively, to obtain a good fit of the data over the whole range of *n*. The parameters *C* and *D* were determined to satisfy the constraint that the function and its derivative are continuous at *n* = ${\bar{n}}$. The obtained *A*, *B*, *C*, *D*, and *p* values, which give high accuracy with sufficiently small root-mean-square deviations from the reference enthalpies (within 27–95 meV), are shown in Table 2.

**Table 2:** Fit parameters for the $\Delta H(n)$ curves [defined in Eq. (12)] for Guinier–Preston (GP1 and GP2) clusters.

Parameter	Value (GP1)	Value (GP2)
${\bar{n}}$	10	40
*A* (eV)	−1.708057	−2.472480
*B* (eV)	−0.164865	−0.192079
*C* (eV)	0.299807	0.647037
*D* (eV)	−0.124505	−0.865633
*p*	0.978498	0.987501

**Figure 1:**
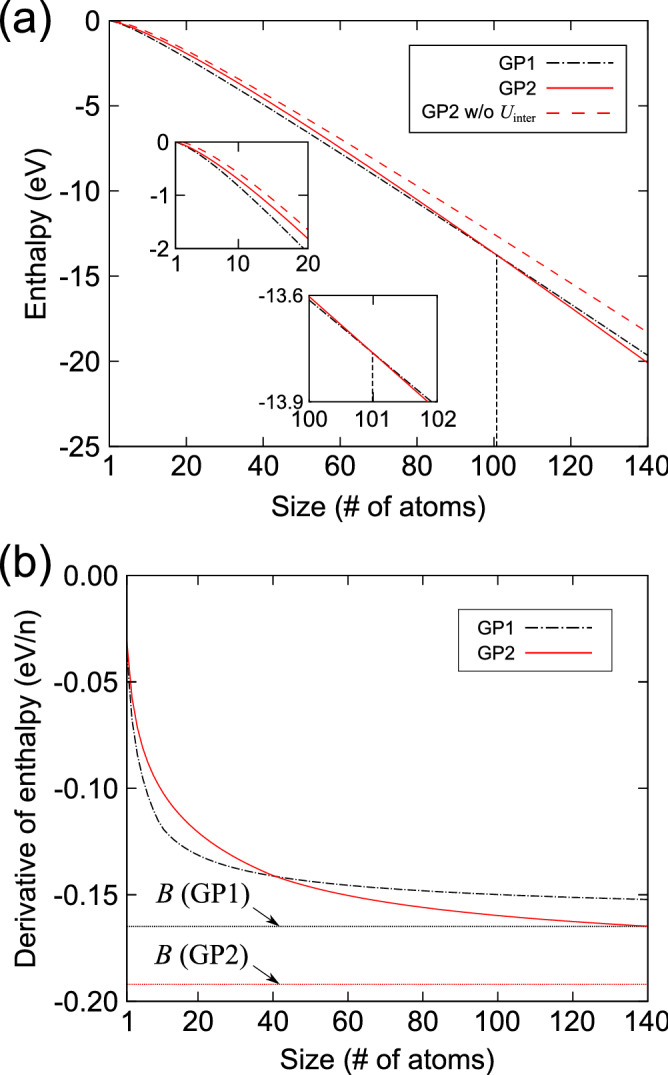
Enthalpies of formation of Guinier–Preston (GP1 and GP2) clusters fit to the function in Eq. (12); (**a**) $\Delta H(n)$ and (**b**) $\partial \Delta H(n)/\partial n$. For comparison, the $\Delta H(n)$ values of GP2 clusters without intercluster interactions ($U^{\text {inter}}$) are shown by the dashed curve. The dotted lines represent the limits of the $\partial \Delta H(n)/\partial n$ values as *n* tends to infinity, corresponding to the parameter *B* in Eq. (12).

Figure 1a shows the curves of the enthalpies of formation of GP1 and GP2 clusters ($\Delta H_{\text {GP1}}(n)$ and $\Delta H_{\text {GP2}}(n)$, respectively) in the range of *n* from 1 to 140. As the *n*-atom GP2 cluster consists of two *n*/2-atom GP1 clusters, its diameter is defined as $1/\sqrt{2}$ times that of the *n*-atom GP1 cluster. Hence, the number of the fully four-coordinated Cu atoms in the central region of the GP2 cluster is smaller than that in the GP1 cluster with the same *n*. As shown in Fig. 1, the $\Delta H_{\text {GP1}}(n)$ is clearly lower than $\Delta H_{\text {GP2}}(n)$ for small *n* values. This indicates that the gain in enthalpy due to the interactions among fully coordinated Cu atoms apparently stabilizes a cluster as its size increases. However, while both $\Delta H_{\text {GP1}}(n)$ and $\Delta H_{\text {GP2}}(n)$ decrease steadily with increasing *n*, the enthalpy of formation of the GP2 cluster falls below that of the GP1 cluster at *n* = 101.0. Eventually, $\Delta H_{\text {GP2}}(n)$ becomes lower than $\Delta H_{\text {GP1}}(n)$ as the size increases. For comparison, the curve of the enthalpy of formation of the GP2 cluster *without* including intercluster interaction energies is also shown in Fig. 1a. It is noteworthy that the enthalpy of formation of the GP2 cluster calculated without considering intercluster interactions is the highest among the three curves for all *n*. This implies that both the multi-body interaction inside the clusters and the intercluster interaction between two Cu platelets significantly contribute to the stabilization of GP clusters.

Figure 1b shows the curves of the derivatives of the enthalpies of formation with respect to *n* (i.e., $\partial \Delta H_{\text {GP1}}(n)/\partial n$ and $\partial \Delta H_{\text {GP2}}(n)/\partial n$). These curves correspond to the energy gain per Cu atom of a growing cluster. Both curves decrease rapidly at small *n*, and then saturate and approach constant values as *n* is increased. While the magnitude of $\partial \Delta H_{\text {GP2}}(n)/\partial n$ is smaller than that of $\partial \Delta H_{\text {GP1}}(n)/\partial n$ at small *n*, the curves cross at $n \approx 40$ and the former falls below the latter at large *n*. This indicates that a certain size is required for the GP2 cluster to be energetically preferable compared with the GP1 cluster owing to the gradual increase in intercluster interactions during the nucleation process.

**Figure 2:**
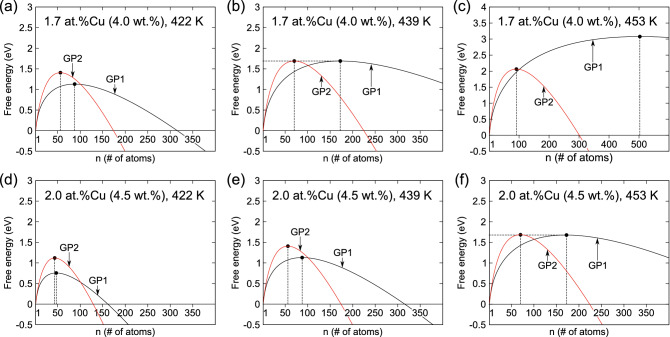
Free energies of formation of Guinier–Preston (GP1 and GP2) clusters in (**a**–**c**) Al-1.7 at%Cu and (**d**–**f**) Al-2.0 at%Cu alloys at temperatures of 422, 439, and 453 K. Note that (**b**) and (**f**) are under conditions where the nucleation barriers of GP1 and GP2 clusters have the same height of 1.69 eV.

### Free energies of formation in the GP1 and GP2 zones

To investigate the nucleation preferences of the GP zones, the free energies of formation for the GP1 and GP2 clusters ($\Delta G_{\text {GP1}}(n)$ and $\Delta G_{\text {GP2}}(n)$, respectively) were obtained using the $\Delta H(n)$ and $\partial \Delta H(n)/\partial n$ curves. Figure 2a–c shows the $\Delta G(n)$ profiles according to Eq. (11) at a Cu concentration of 1.7 at% (4.0 wt%) at various temperatures. The nucleation barriers (i.e., the maximum free energy of formation at the critical nucleus size) of the GP1 and GP2 zones steadily increase with increasing temperature; moreover, the magnitude ratios of these values clearly change with temperature.

The GP1 and GP2 zones nucleate with equal probability under the condition that their nucleation barriers are balanced, if there is no significant difference between the corresponding values of both prefactors. Based on Eq. (11), we found that the relation $\Delta G_{\text {GP1}}(n^{*})$ = $\Delta G_{\text {GP2}}(n^{*})$ holds only at $n^{*}_{\text {GP1}}$ = 172.4 and $n^{*}_{\text {GP2}}$ = 70.1, which results in a unique value of the nucleation barrier of 1.69 eV. Importantly, the possible combinations of temperature and Cu concentration satisfying the above condition can vary according to Eq. (4) or (10). In the case of 1.7 at% Cu in the Al alloy, $\Delta G_{\text {GP1}}(n^{*})$ and $\Delta G_{\text {GP2}}(n^{*})$ are balanced at 439 K, as shown in Fig. 2b. This indicates that the relationship between the nucleation rates of the two GP zones is reversed at 439 K; GP1 and GP2 clusters nucleate preferentially below and above 439 K, respectively. A brief discussion of the non-ideal configurational contributions to the $\Delta G(n)$ curves is provided in the Supplementary Information. Hardy^9,10^ and Hirano^12^ investigated the incubation periods of GP zones (i.e., the aging times required to begin the formation of GP zones) as a function of the aging temperature in an Al-1.7 at%Cu alloy using hardness aging curves and thermal measurements, respectively. They reported that the incubation times of the GP1 and GP2 formation coincide at 438–442 K, which agrees well with our result of 439 K.

Similar relationships between the nucleation barriers of GP1 and GP2 zones were observed with increasing (decreasing) Cu concentration, and with decreasing (increasing) temperature. Figure 2d–f shows the $\Delta G(n)$ curves for 2.0 at% (4.5 wt%) Cu at various temperatures. By comparing the upper and lower panels, it can be seen that the nucleation barriers of the GP1 and GP2 zones steadily decrease with increasing Cu concentration at constant temperature. In fact, the curves in Fig. 2a and e, and those in Fig. 2b and f are identical, whereas the temperature and concentration conditions differ according to Eq. (4) or (10).

In Fig. 2a–c, the free energy of formation profiles of the two GP zones cross at $n = 101.0$, which is ascribed to the crossover of $\Delta H(n)$, as in Fig. 1a, since we assume the same form of $\Delta S(n)$ for the GP1 and GP2 clusters. When $n^{*}_{\text {GP1}}$ < 101.0 at low temperatures, as in Fig. 2a, the GP1 cluster nucleates preferentially owing to its low energy barrier; then, it is expected to transform into the GP2 structure for sizes of > 101.0 and settle into a lower free-energy state. However, when $n^{*}_{\text {GP1}}$ > 172.4 at high temperatures, as in Fig. 2c, the GP2 zones can directly nucleate and grow without the initial growth of GP1 zones. The diameter of a GP1 cluster at *n* = 101.0 is approximately 4 nm, which is consistent with the average experimental values of GP1-zone diameters^7,11,13–15,23,24^. This implies that the saturation of the size of GP1 zones with aging time is associated with the crossover of the free energies of formation at $n = 101.0$, above which GP zones start to grow again as GP2 zones.

**Figure 3:**
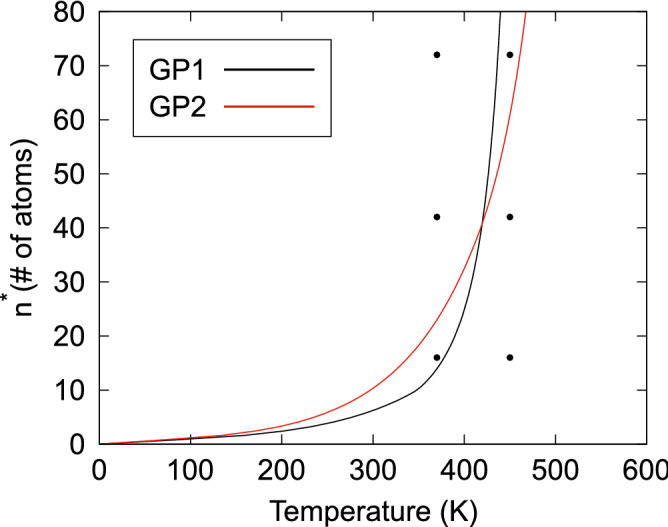
Critical nucleus sizes of Guinier–Preston (GP1 and GP2) clusters as a function of temperature at a Cu concentration of 2.0 at%. The plots represent the cases selected for the kinetic Monte Carlo (kMC) calculations for cluster sizes of 16, 42, or 72 atoms and temperatures of 370 or 450 K.

### Verification by atomistic kinetic Monte Carlo calculations

Figure 3 shows the critical nucleus sizes of GP1 and GP2 clusters at a Cu concentration of 2.0 at% predicted by the CNT model. As described in the previous sections, clusters with a size above that of the critical nucleus tend to grow spontaneously, whereas those below this size tend to shrink. To examine the CNT results of the temperature dependence of critical nucleus sizes from a kinetic point of view, we used atomistic kinetic Monte Carlo (kMC) calculations^29^, which can describe the effect of configurational entropy without using the approximation based on the ideal solution model given in Eq. (3).

We used a $20a_{\text {Al}}\times 20a_{\text {Al}}\times 10a_{\text {Al}}$ supercell of fcc Al containing 16000 lattice points, where $a_{\text {Al}}$ is the lattice constant of fcc Al. As initial configurations, one GP1 or GP2 cluster consisting of 16, 42, or 72 Cu atoms was constructed and embedded in the supercell; residual Cu atoms (and one vacancy) were randomly distributed while maintaining a composition of Al-2.0 at%Cu-0.00625 at%vacancy. In total, 20 different initial configurations of supercells were prepared for each case. We systematically conducted atomistic kMC calculations of growth (or shrinkage) of GP1 and GP2 clusters at temperatures of 370 and 450 K using the same potential applied to the CNT model. It should be noted that vacancy-solute interactions and the activation barriers for vacancy migration were considered following our previous study^29^.

**Table 3:** Probability of growth of the embedded Cu cluster during 20 runs of a kinetic Monte Carlo (kMC) calculation.

Size	Temperature	
	370 K	450 K
**GP1 cluster**		
72 atoms	100% ($\nearrow$)	50% ($\rightarrow$)
42 atoms	100% ($\nearrow$)	5% ($\searrow$)
16 atoms	75% ($\nearrow$)	5% ($\searrow$)
**GP2 cluster**		
72 atoms	100% ($\nearrow$)	100% ($\nearrow$)
42 atoms	100% ($\nearrow$)	50% ($\rightarrow$)
16 atoms	20% ($\searrow$)	0% ($\searrow$)

Northeast and southeast arrows inside parentheses represent cases where the probability is > 50% and < 50%, respectively.

As shown in Fig. 3, for the GP1 clusters, the CNT results suggest that three clusters of different sizes tend to grow at 370 K, while they tend to shrink at 450 K. In contrast, for the GP2 clusters, the 72-atom clusters at both temperatures and 42-atom clusters at 370 K tend to grow. Table 3 summarizes the probabilities of growth of the GP1 and GP2 clusters by counting the cases in which the size of the embedded cluster increases after $5\times 10^{6}$ steps in the kMC calculations. Overall, the kMC results agree well with the CNT results, although the cases of 72-atom GP1 and 42-atom GP2 clusters at 450 K do not show a clear trend over the limited number of calculation steps. Consequently, the results indicate that the present atomistic kMC and CNT models are consistent with each other. Hence, the interplay between enthalpic and entropic effects are adequately described by our CNT model for determining the critical nucleus size of GP1 and GP2 clusters.

**Figure 4:**
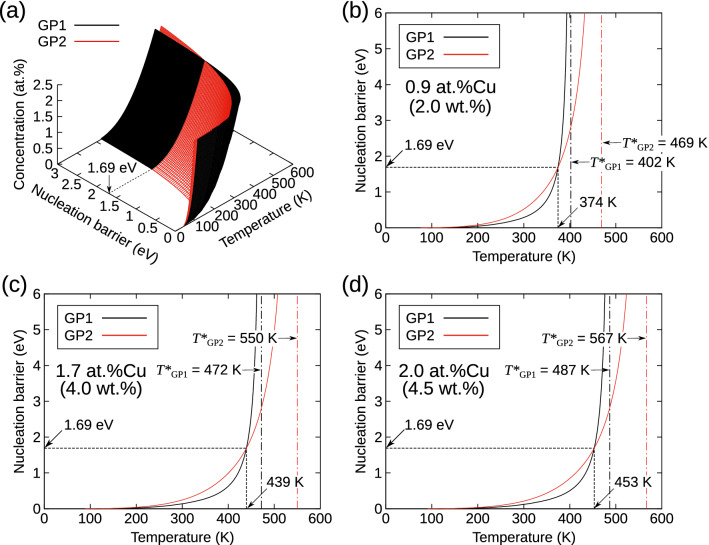
Results from the classical nucleation theory (CNT)-based model for the nucleation barriers of Guinier–Preston (GP1 and GP2) clusters in Al–Cu alloys as a function of temperature and Cu concentration. (**a**) Temperature-concentration diagram of the nucleation barriers and the cross-sections at specific Cu concentrations of (**b**) 0.9, (**c**) 1.7, and (**d**) 2.0 at%. The vertical dash-dotted lines represent the upper limit temperatures ($T^{*}$) for the formation of GP zones.

### Effects of temperature and Cu concentration on nucleation barriers of GP zones

Figure 4a shows a temperature-concentration diagram of the nucleation barriers ($\Delta G(n^{*})$) of GP1 and GP2 zones, indicating the nucleation preferences of the two cluster types at a given temperature and Cu concentration. The temperature dependence of $\Delta G(n^{*})$ at a specific Cu concentration was obtained by extracting cross-sections of the diagram, as shown in Fig. 4b–d. The $\Delta G(n^{*})$ increases with temperature, gradually at low temperature and then much more rapidly at high temperature. Here, we define $T_{\text {c}}$ as the crossover temperature where the nucleation barriers of the GP1 and GP2 clusters are equal. The GP1 and GP2 clusters nucleate preferentially over the other below and above $T_{\text {c}}$, respectively. Note that $\Delta G(n^{*})$ at $T_{\text {c}}$ is always 1.69 eV, as described in the previous section, while $T_{\text {c}}$ increases with increasing Cu concentration (i.e., $T_{\text {c}}$ = 374, 439, and 453 K at 0.9, 1.7, and 2.0 at%, respectively).

Furthermore, it should be noted that $\Delta G(n^{*})$ diverges to infinity with increasing temperature; thus, the upper temperature limit ($T^{*}$) is defined as the temperature above which GP1 or GP2 clusters cannot survive at a given Cu concentration. In our model, the $T^{*}$ values were derived by taking the limit $\partial \Delta H(n)/\partial n\rightarrow B$ as $n\rightarrow \infty$ in Eq. (4) or (10). For example, in the case of an Al-1.7 at%Cu alloy, as shown in Fig. 4c, GP1 and GP2 clusters of any size become unstable and tend to shrink above $T^{*}$ values of 472 and 550 K, respectively.

**Figure 5:**
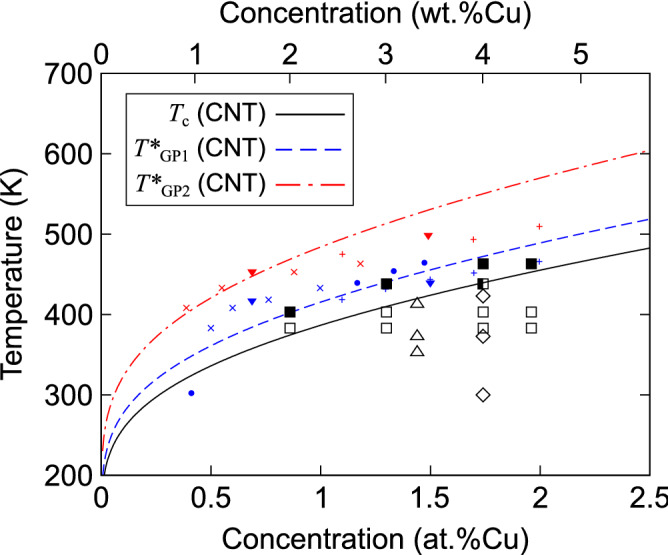
Results from the classical nucleation theory (CNT)-based model for the crossover temperature ($T_{\text {c}}$) and upper limit temperature ($T^{*}$) of Guinier–Preston (GP1 and GP2)-zone formation as a function of Cu concentration. The $T_{\text {c}}$ (solid curve) denotes the temperature at which the nucleation barriers of GP1 and GP2 clusters are equivalent. The $T^{*}_{\text {GP1}}$ (dashed curve) and $T^{*}_{\text {GP2}}$ (dash-dotted curve) represent the temperatures beyond which GP1 and GP2 zones, respectively, cannot exist for a given Cu concentration. The experimental data for the structures first detected during the aging process are taken from Refs.^7,11,49^; open and solid squares (GP1 and GP2, respectively)^11^, diamonds (GP1)^7^, and triangles (GP1)^49^. The experimental data for the solvi of the GP zones are taken from Refs.^39–42^; plus symbols (GP1 and GP2)^39^, crosses (GP1 and GP2)^40^, inverted triangles (GP1 and GP2)^41^, and circles (GP1)^42^. Plots of the GP1 and GP2 solvi are colored in blue and red, respectively.

Consistent with experimental results, the obtained $T^{*}$ values should correspond to the solvus temperature of GP zones^39–42^. It should be noted that, as in Fig. 4, $T_{\text {c}}$ is always lower than the $T^{*}$ of GP1 zones. This implies that the nucleation preferences of the GP1 and GP2 zones can change in the temperature range lower than their solvus temperatures. To verify this point, the Cu-concentration dependence of $T_{\text {c}}$ and $T^{*}$ was investigated and compared with experimental results, as shown in Fig. 5. The large and small plots represent the structures first detected during the aging process^7,11,15^ and the solvi of the GP zones^39–42^, respectively, in Al–Cu alloys. It is worth noting that our predicted $T_{\text {c}}$ curve clearly marks the boundary between the GP1 and GP2 nucleation. In addition, the obtained $T^{*}$ values of the GP1 and GP2 zones are in good agreement with the experimental counterparts. The similarity between these values should not be overemphasized because of the approximation of the free energy of formation values in the CNT-based model. However, this indicates that the nucleation preference of the single- and double-layer GP zones is governed by the crossover between their enthalpies of formation with increasing cluster size, which arises from the competing interactions within and between the clusters.

## Conclusions

Using the CNT model, we revealed the competing nucleation mechanisms between the GP1 and GP2 zones in Al–Cu alloys, which have hitherto not been either experimentally or theoretically clarified. The precipitation sequence of the two GP zones was assessed by constructing and exploring a temperature-concentration diagram of the nucleation barriers of the GP1 and GP2 zones. We predict two pathways for the formation of GP2 zones. When the nucleation barrier of the GP1 zones is smaller than that of the GP2 zones at low temperatures and/or high Cu concentration, GP1 zones preferentially nucleate but stop growing with aging since they become less energetically favorable than GP2 zones as the zone size increases. The upper size limit of GP1 zones is determined by the crossover of the free energies (or enthalpies) of formation at $n = 101$, which corresponds to a diameter of approximately 4 nm. GP zones start to grow again after transformation from the GP1 to the GP2 structure for sizes of > 101. Such a crossover results in the discontinuous growth process of GP zones. In contrast, when the nucleation barrier of the GP2 zones is smaller than that of the GP1 zones, which is predicted to occur at high temperatures (i.e., above the crossover temperature) and/or at low Cu concentration, GP2 zones can nucleate directly without the induction of GP1 zones. These results provide a coherent explanation of the effects of temperature and Cu concentration on the nucleation of GP zones in Al–Cu alloys. In addition, our analyses show good correspondence with experimental observations of GP zones at various temperatures and Cu concentrations. We anticipate that our findings will serve as a basis for the development of novel heat-treatment strategies to efficiently enhance the strength of alloys by controlling the growth of particular solute-rich zones.

## Methods

### Potential for GP zones in dilute Al–Cu alloys

We describe the on-lattice potential of a dilute Al–Cu system *U* as an approximation of the energy of formation $E_{\text {f}}$:

13 $$\begin{aligned} E_{\text {f}}\approx & {} U(\{{\varvec{r}}_i\}) \nonumber \\= & {} U^{\text {2body}}+U^{\text {3body}}+U^{\text {mbody}}+U^{\text {inter}}, \end{aligned}$$

where ${\varvec{r}}_{i}$ is the coordinates of atom *i* in the system; and $U^{\text {2body}}$, $U^{\text {3body}}$, and $U^{\text {mbody}}$ are the two-body, three-body, and multi-body interaction energies, respectively. In the present model, the main contribution to the total energy of the system (i.e., bonding and cohesion) comes from the sum of the two-body energies ($U^{\text {2body}}$), where $U^{\text {3body}}$ and $U^{\text {mbody}}$ are introduced as correction terms to the two-body additive approximation for the energy of formation. For detailed explanation on the formalism and parameterization of $U^{\text {2body}}$, $U^{\text {3body}}$, and $U^{\text {mbody}}$, please refer to our previous work^29^.

The last term $U^{\text {inter}}$ in Eq. (13) was introduced to describe the energy of interaction between the plate-like GP1 clusters (i.e., intercluster energy) at a given distance along the out-of-plane direction. For details on the potential parameterization, see the later section. The potential was used to determine the energetics of the GP1 and GP2 clusters in the dilute Al–Cu system, while reducing computational costs and maintaining the accuracy close to that of DFT calculations.

It should be noted that the vibrational contribution to the free energies of formation, including the zero-point energy (ZPE) correction, was not considered in our calculations. To estimate the vibrational contributions to the free energies, the ZPE and vibrational free energy of the configuration of a Cu–Cu–Cu triplet were calculated, and the binding energies with and without the vibrational contribution were compared (see Supplementary Information). These results suggested that the vibrational contribution does not significantly change the binding nature of the Cu–Cu–Cu triplet. As a trade-off between the accuracy of the calculations and computational feasibility, we used the energies without the ZPE correction in this study.

### Atomic structures of GP zones

The enthalpy of formation values for GP1 and GP2 Cu clusters at 0 K were calculated using the proposed potential. The reference state was defined as the set of isolated (infinitely separated) Cu atoms in the matrix (i.e., $\Delta H(1) = 0$). The GP1 cluster was modeled as a single-layer, square-shaped cluster, whose structure was determined such that Cu atoms were spirally arranged one by one on a specific $\{100\}$ plane, as shown in Supplementary Fig. S1 online. Note that the Cu atoms in the central area of the cluster are bound by four Cu atoms at the first-nearest neighbor (1NN) distance, while those near the edge are less coordinated. This configuration gave the minimum enthalpy of formation among all of the structures with the same *n*, as the maximum number of Cu atoms with a high coordination number was obtained. The GP2 *n*-atom cluster was modeled as two GP1 clusters, each consisting of *n*/2 solute atoms, separated by a distance of 2$a_{\text {Al}}$.

### Intercluster interactions between GP zones in dilute Al–Cu alloys

To calculate the enthalpies of formation of the GP1 and GP2 clusters in the Al–Cu system with DFT accuracy, we extended a previously developed potential^29^ by adding a contribution of intercluster interaction based on the DFT-calculated energies^33^. While the GP2 zones in the Al–Cu system may consist of two or more Cu layers separated by a few (one to three) Al layers^7,8^, the typical structure consists of two Cu platelets separated by 2$a_{\text {Al}}$. Similar tendencies of short-range and long-range ordering on nanometer scales have been observed in other alloy systems, such as Mg-Y, Mg-Zn-Y, Au-Ni, and Ni-Mo^43–48^.

Nakagami et al.^33^ described the energetics of GP2 clusters by introducing an intercluster energy of interaction between two Cu platelets. Supplementary Fig. S2a online shows the atomic structure of a GP2 cluster, in which the black spheres (connected by chain lines as a guide to the eye) represent a pair of Cu atoms separated by 2$a_{\text {Al}}$ in the direction normal (out-of-plane) to the disk-shaped clusters. To characterize the interaction between the clusters at the atomic level, the 1NN coordination number of Cu atoms on the {100} planes are distinguished (from 0 to 4 as in Supplementary Fig. S2b online) and used to describe the change in the strength of the intercluster energy of interaction between the Cu atoms at the center and edge of clusters as a function of the coordination number. Based on the work by Nakagami et al.^33^, $U^{\text {inter}}$ was defined as the sum of the energies of interaction between the Cu atoms in the form:

14 $$\begin{aligned} U^{\text {inter}}= & {} \frac{1}{2}\sum ^{N}_{j\ne i}\sum ^{4}_{\alpha =0}\sum ^{4}_{\beta =0}\sum _{\lambda \in \langle 100\rangle } \epsilon ^{(\alpha )(\beta )}_{\text {inter}} \nonumber \\&\times \delta ({\varvec{r}}_{ij}-2a_{\text {Al}}{\varvec{e}}_{\lambda })\delta (c_i-\alpha )\delta (c_j-\beta ), \end{aligned}$$

where $\epsilon ^{(\alpha )(\beta )}_{\text {inter}}$ is the intercluster energy of the interaction depending on the coordination numbers $\alpha$ and $\beta$ $(0\le \alpha ,\beta \le 4)$; ${\varvec{r}}_{ij}$ is the distance vector between atoms *i* and *j*; ${\varvec{e}}_{\lambda }$ is the unit vector parallel to the $\langle$100$\rangle$ directions (i.e., $\{\lambda$ | [100], $[{\bar{1}}00]$, [010], $[0{\bar{1}}0]$, [001], $[00{\bar{1}}]\}$); $c_{i}$ is the number of neighboring Cu atoms around atom *i* on the $\{100\}$ plane; *N* is the number of Cu atoms; and $\delta$ is the Dirac delta function.

Parameterization of $U^{\text {inter}}$ was performed based on the DFT results of the energies of formation of GP2 clusters in the Al–Cu system^33^. The obtained parameters for intercluster energies of interaction are listed in Supplementary Table S1 online.

## Supplementary information

Supplementary Information.

## Data Availability

The data that support the findings within this paper are available from the corresponding author upon reasonable request.
