# Supplementary material for: Competing nucleation of single- and double-layer Guinier–Preston zones in Al–Cu alloys
Source: Sci Rep. 2021 Feb 24;11:4503. doi: 10.1038/s41598-021-83920-8 (PMC7904818; doi:10.1038/s41598-021-83920-8)
Supplement: Supplementary file 1 — Supplementary Information. [file 41598_2021_83920_MOESM1_ESM.pdf]

Supplementary Information

Competing nucleation of single- and double-layer Guinier-Preston zones in Al-Cu alloys

Hiroshi Miyoshi,[1] Hajime Kimizuka,[2,*] Akio Ishii,[1] and Shigenobu Ogata[1,3]

[1] *Department of Mechanical Science and Bioengineering, Graduate School of Engineering Science, Osaka University, Osaka 560-8531, Japan*

[2] *Department of Materials Design Innovation Engineering, Graduate School of Engineering, Nagoya University, Aichi 464-8603, Japan*

[3] *Center for Elements Strategy Initiative for Structural Materials (ESISM), Kyoto University, Kyoto 606-8501, Japan*

* kimizuka@nagoya-u.jp (Corresponding author)

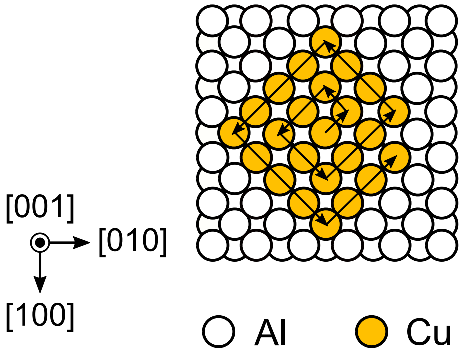

Supplementary Figure S1. Schematic of the arrangement of solute atoms composing a single-layer Guinier-Preston (GP1) cluster along the (001) plane in the fcc lattice. Al and Cu atoms are displayed in open (white) and solid (yellow) circles, respectively.

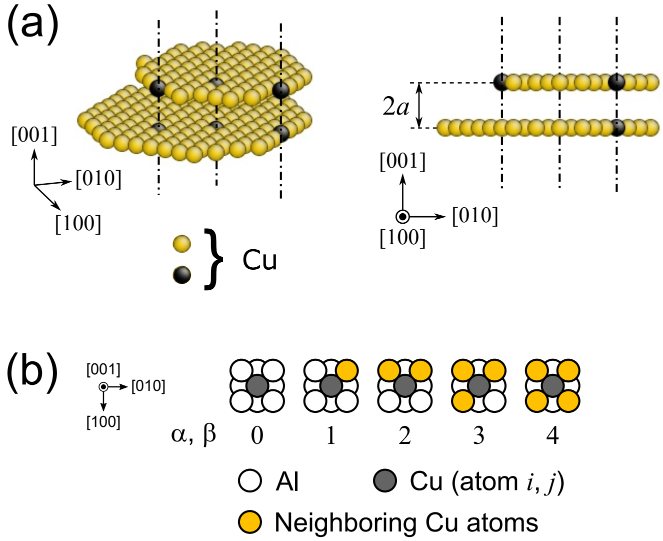

Supplementary Figure S2. Atomic structure model of the Guinier-Preston (GP) zones. (a) An example of atomic arrangements of a double-layer GP (GP2) cluster consisting of two single-layer GP (GP1) clusters separated by $2a_{\mathrm{Al}}$. (b) Definition of the first nearest neighbor (1NN) coordination numbers of Cu atoms on the $\{100\}$ planes ($0 \leq \alpha, \beta \leq 4$).

Supplementary Table S1. Intercluster energies of interaction ($\epsilon_{\text{inter}}^{(\alpha)(\beta)}$ in meV) as a function of the coordination numbers α and β.

β	α				
	0	1	2	3	4
0	0	0	0	0	−5
1	0	0	0	−3	−9
2	0	0	0	−8	−14
3	0	−3	−8	−14	−21
4	−5	−9	−14	−21	−29

A. Comparison of models for the configurational entropy

To examine the non-ideal configurational contribution to the free energies, we calculated the configurational entropy for the dispersed cluster system where the solute atoms of concentration x are clustered only by the amount x_c, while $(x - x_c)$ remains unclustered in solution [1]. In this case, the clusters with the size n and concentration x_c are distributed throughout the entire system, while the remnant solute atoms of the concentration $(x - x_c)$ are scattered throughout the remaining space. The entropy change (per cluster) from the original solution state when the clusters are formed is expressed as:

$$\Delta S = -k_B \left[\ln\left(\frac{x_c}{n\xi}\right) + n\left(\frac{x}{x_c} - 1\right)\ln(x - x_c) - n\left(\frac{x}{x_c}\right)\ln x + (n-1) \right], \tag{S1}$$

where ξ represents the shape diversity of the cluster, and corresponds to W_{rot} for the GP1 and GP2 clusters. Further, x_c is given by $x_c = nC(n) = nx\exp(-\Delta G(n)/k_B T)$ [2].

Figure S3 shows the free energies of formation for the GP1 and GP2 clusters ($\Delta G_{\text{GP1}}(n)$ and $\Delta G_{\text{GP2}}(n)$, respectively) at a Cu concentration of 1.7 at.%, obtained from the entropies of formation according to the ideal solution model (equation (3) in the main text) and the dispersed cluster model (equation (S1)). For both the GP1 and GP2 clusters, the nucleation barriers ($\Delta G(n^*)$) obtained from the dispersed cluster model are about half the height of those obtained from the ideal solution model at the same temperature. This is because the dispersed cluster model considers the increase in configurational entropy associated with the cluster distribution in the system. It should be noted that the critical nucleus size (n^*) values obtained from both models are equal, and the relationship between the nucleation barriers of the two GP zones is maintained, such that the relation $\Delta G_{\text{GP1}}(n^*) = \Delta G_{\text{GP2}}(n^*)$ holds at 439 K. This implies that the crossover temperatures (T_c) for the nucleation of GP1 and GP2 clusters predicted in this study are the same with either model. Furthermore, in the dispersed cluster model, the condition corresponding to equation (4) in the main text is described as:

$$\left.\frac{\partial \Delta H(n)}{\partial n}\right|_{n=n^*} = k_B T \ln(x - x_c|_{n=n^*}). \tag{S2}$$

Note that it becomes equivalent to equation (4) when $x_c \approx 0$ for sufficiently large n^* and $\Delta G(n^*)$. This indicates that the upper temperature limits (T^*) for the formation of GP clusters are also the same with either model. Thus, our CNT model using the configurational entropy based on the ideal solution model gives robust results for this problem.

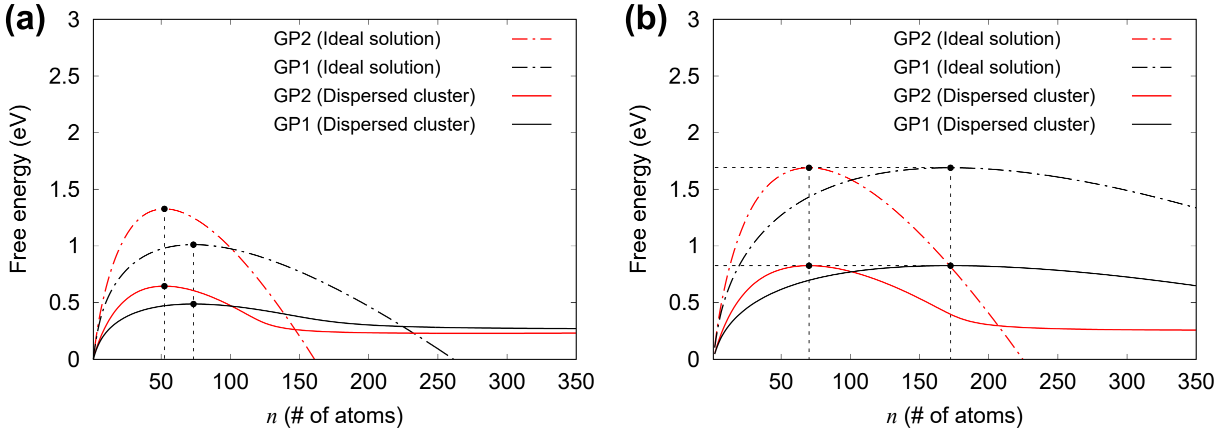

Supplementary Figure S3. Free energies of formation of Guinier-Preston (GP1 and GP2) clusters in an Al-1.7 at.%Cu alloy at temperatures of (a) 422 and (b) 439 K. The results are obtained from the entropies of formation of an n-atom solute cluster according to the ideal solution model (dash-dotted curves) and the dispersed cluster model (solid curves). Note that (b) is under a condition where the nucleation barriers of GP1 and GP2 clusters have the same height.

B. Vibrational contribution to the binding energy of solute atoms

To examine the vibrational contribution to the free energies, we calculated the zero-point energy (ZPE) and vibrational free energy of the configuration of a Cu-Cu-Cu triplet with a bond angle of 90°, which is one of the main components leading to the formation of GP clusters. The Hessian matrix and vibrational frequencies of the corresponding systems were determined from the forces acting on atoms using DFT calculations, in which each atom was displaced (by ±0.01 Å) in the direction of each Cartesian coordinate. Based on the quantum statistics in the harmonic approximation, vibrational free energies are described as

$$F_{\mathrm{vib}} = \sum_i \left\{ \frac{1}{2} h\nu_i + k_{\mathrm{B}}T \ln \left[1 - \exp \left(-\frac{h\nu_i}{k_{\mathrm{B}}T} \right) \right] \right\}, \tag{S3}$$

where ν_i is the vibrational frequency of the ith normal mode and h is Planck's constant. The obtained ZPE-corrected binding energy and binding free energy at 300 K were −0.06 eV and −0.05 eV, respectively, while the DFT result without the ZPE correction was −0.07 eV. This suggests that the vibrational contribution does not significantly change the binding nature of the Cu-Cu-Cu triplet, whereas its binding strength tends to reduce with increasing temperature.

Since further examination of the vibrational contribution requires an extensive and careful study, it will be the subject of future work. As a trade-off between the accuracy of the calculations and computational feasibility, we used the energies without ZPE correction in this study.

---

[1] Fujita, F. E. On the small atomic clusters dispersed in solids. *Mater. Trans., JIM* **38**, 659–667 (1997).

[2] Toschev, S. Homogeneous nucleation. In Hartman, P. (ed.) *Crystal Growth: An Introduction*, 1–49 (North-Holland, 1973).
